# Clinical impact of vivax malaria: A collection review

**DOI:** 10.1371/journal.pmed.1003890

**Published:** 2022-01-18

**Authors:** Aung Pyae Phyo, Prabin Dahal, Mayfong Mayxay, Elizabeth A. Ashley

**Affiliations:** 1 Myanmar Oxford Clinical Research Unit, Yangon, Myanmar; 2 Infectious Diseases Data Observatory–IDDO, Oxford, United Kingdom; 3 Centre for Tropical Medicine and Global Health, Nuffield Department of Medicine, University of Oxford, Oxford, United Kingdom; 4 Institute of Research and Education Development (IRED), University of Health Sciences, Ministry of Health, Vientiane, Laos; 5 Lao–Oxford–Mahosot Hospital–Wellcome Trust Research Unit, Mahosot Hospital, Vientiane, Laos; Burnet Institute, AUSTRALIA

## Abstract

**Background:**

*Plasmodium vivax* infects an estimated 7 million people every year. Previously, vivax malaria was perceived as a benign condition, particularly when compared to falciparum malaria. Reports of the severe clinical impacts of vivax malaria have been increasing over the last decade.

**Methods and findings:**

We describe the main clinical impacts of vivax malaria, incorporating a rapid systematic review of severe disease with meta-analysis of data from studies with clearly defined denominators, stratified by hospitalization status. Severe anemia is a serious consequence of relapsing infections in children in endemic areas, in whom vivax malaria causes increased morbidity and mortality and impaired school performance. *P*. *vivax* infection in pregnancy is associated with maternal anemia, prematurity, fetal loss, and low birth weight. More than 11,658 patients with severe vivax malaria have been reported since 1929, with 15,954 manifestations of severe malaria, of which only 7,157 (45%) conformed to the World Health Organization (WHO) diagnostic criteria. Out of 423 articles, 311 (74%) were published since 2010. In a random-effects meta-analysis of 85 studies, 68 of which were in hospitalized patients with vivax malaria, we estimated the proportion of patients with WHO-defined severe disease as 0.7% [95% confidence interval (CI) 0.19% to 2.57%] in all patients with vivax malaria and 7.11% [95% CI 4.30% to 11.55%] in hospitalized patients. We estimated the mortality from vivax malaria as 0.01% [95% CI 0.00% to 0.07%] in all patients and 0.56% [95% CI 0.35% to 0.92%] in hospital settings. WHO-defined cerebral, respiratory, and renal severe complications were generally estimated to occur in fewer than 0.5% patients in all included studies. Limitations of this review include the observational nature and small size of most of the studies of severe vivax malaria, high heterogeneity of included studies which were predominantly in hospitalized patients (who were therefore more likely to be severely unwell), and high risk of bias including small study effects.

**Conclusions:**

Young children and pregnant women are particularly vulnerable to adverse clinical impacts of vivax malaria, and preventing infections and relapse in this groups is a priority. Substantial evidence of severe presentations of vivax malaria has accrued over the last 10 years, but reporting is inconsistent. There are major knowledge gaps, for example, limited understanding of the underlying pathophysiology and the reason for the heterogenous geographical distribution of reported complications. An adapted case definition of severe vivax malaria would facilitate surveillance and future research to better understand this condition.

## Abstract 2

Elizabeth A Ashley and colleagues describe the main clinical impacts of vivax malaria globally.

## Author summary

**Why was this study done?:**

- Historically, the clinical impact of the relapsing malaria caused by *Plasmodium vivax* has been understudied and reported compared to falciparum malaria.

**What did the researchers do and find?:**

- We reviewed the literature on the clinical impact of vivax malaria focusing on children and pregnant women and performed a rapid systematic review of published evidence for severe disease and meta-analysis of selected studies.
- Vivax malaria in young children is associated with severe anemia and increased mortality. Infections in pregnancy lead to maternal anemia, prematurity, fetal loss, and low birth weight. We estimated the proportion of patients with World Health Organization (WHO)-defined severe disease as 0.7% [95% confidence interval (CI) 0.19% to 2.57%] in all patients with vivax malaria and the mortality from vivax malaria as 0.01% [95% CI 0.00% to 0.07%] in all patients and 0.56% [95% CI 0.35% to 0.92%] in hospital settings.

**What do these findings mean?:**

- Reporting of severe vivax malaria has increased dramatically over the last 10 years, but different case definitions have been applied.
- Limitations of our analysis include high risk of bias of published studies, the majority of which are in hospitalized patients (who are therefore more likely to be severely unwell), while most vivax malaria is managed in primary care. As a result, estimates should be interpreted with caution.
- Prospective cohort studies using an adapted case definition of severe vivax malaria would improve estimates of incidence.
- Preventing vivax malaria and subsequent relapses in children and pregnant women is a priority to reduce morbidity and mortality.

## Introduction

Like *Plasmodium falciparum*, the *Plasmodium vivax* parasite has an intraerythrocytic life cycle lasting approximately 48 hours from red cell invasion by merozoites to schizont rupture. In synchronous infections, schizogony triggers a febrile paroxysm every 3 days, hence the term “tertian fever” given to early descriptions of patients with vivax or falciparum malaria. Malaria caused by *P*. *vivax* was termed benign tertian malaria because the clinical course was generally considered to be mild compared to falciparum malaria even though occasional descriptions of severe, including cerebral, presentations of vivax malaria have been reported since 1902 [1]. The notion that *P*. *vivax* only rarely causes severe malaria has been challenged over the last 15 years [2], and reports of severe disease have been increasing. More evidence for the negative impacts of vivax malaria on young children and pregnant women has emerged over the same time period. Here, we describe the clinical symptoms and major clinical impacts of malaria caused by *P*. *vivax* taking a narrative approach but incorporating a rapid systematic review of the evidence for severe disease.

## Rapid review methods

We searched PubMed for articles in English (including those with an English abstract only) documenting severe vivax malaria published until August 1, 2021, using a variety of terms to search title and abstract (S1 Table). Articles were screened by one reviewer (APP) with 20% screened by a second reviewer (PD or EAA). Extra references were identified from other systematic reviews, bibliographic searches, and searching in Google Scholar. This review is reported as per the Preferred Reporting Items for Systematic Reviews and Meta-Analyses (PRISMA) guideline (S2 Table).

### Definitions

We included articles if the authors classified the episode as severe vivax malaria. We noted whether the cases met the case definition of severe (falciparum) malaria published by the World Health Organization (WHO) in 2014 [3], without taking parasite density into account in the definitions of jaundice or anemia (Table 1).

**Table 1:** WHO definition of severe falciparum malaria and common adaptations used to report severe *Plasmodium vivax* malaria.

Criterion	Definition for *P. falciparum*	Criteria used to define patients diagnosed with severe *P. vivax* malaria included in the review
Impaired consciousness	GCS <11 in adults or Blantyre coma score <3 in children	Obtunded, drowsy, GCS 11 to 15 or not specified
Acidosis	Base deficit of >8 meq/l or, if unavailable, a plasma bicarbonate of <15 mM or venous plasma lactate >5 mM. Severe acidosis manifests clinically as respiratory distress—rapid, deep, and labored breathing	Same definition
Hypoglycemia	Blood or plasma glucose <2.2 mM (<40 mg/dl)	Same cutoff but a few articles use 60 mg/dl threshold
Severe malarial anemia	Hb concentration <5 g/dl or a hematocrit of <15% in children <12 years of age (<7 g/dl and <20%, respectively, in adults) together with a parasite count >10,000/μL	Other cutoff such as <8, 6, or 9 g/dl
AKI	Plasma or serum creatinine >265 μM (3 mg/dl) or blood urea >20 mM	KDIGO or RIFLE staging, different urea and creatinine cutoffs
Jaundice	Plasma or serum bilirubin >50 μM (3 mg/dl) together with a parasite count >100,000/μL	Clinical jaundice or different bilirubin cutoffs. No parasite density requirement
Pulmonary edema	Radiologically confirmed or oxygen saturation <92% on room air with a respiratory rate >30/min, often with chest indrawing and crepitations on auscultation	ARDS (PaO2/FiO2 ratio cutoff 300 or unquantified), pleural effusion or acute lung injury (unspecified)
Significant bleeding	Including recurrent or prolonged bleeding from nose, gums or venepuncture sites; hematemesis or melena	Various forms of bleeding ranging from petechia and epistaxis to disseminated intravascular coagulation. Thrombocytopenia included, categorized using different platelet count thresholds of 20, 35, 50, and 60 × 10^9^/L
Shock	Compensated shock is defined as capillary refill ≥3 s or temperature gradient on leg (mid to proximal limb), but no hypotension. Decompensated shock is defined as SBP <70 mm Hg in children or <80 mm Hg in adults with evidence of impaired perfusion (cool peripheries or prolonged capillary refill)	Different SBP cutoff 70 mm Hg, 80 mm Hg, or mean BP not specified in some cases
Hyperparasitemia	*P*. *falciparum* parasitemia >10% parasitized erythrocytes	Different parasitemia cutoffs such as 50,000/ μL, 100,000/μL, >4% to 5% or unquantified

AKI, acute kidney injury; ARDS, acute respiratory distress syndrome; GCS, Glasgow Coma Score; Hb, hemoglobin; KDIGO, Kidney Disease: Improving Global Outcomes; RIFLE, Risk, Injury, Failure; Loss, End-Stage renal disease; SBP, systolic blood pressure; WHO, World Health Organization.

### Statistical analysis

The proportion of patients with severe vivax malaria was estimated using a meta-analysis of single proportions using data from studies with clearly defined denominators (case reports, case series, and studies exclusively in pregnant women were excluded), after applying logit transformation. Heterogeneity was assessed using the *I*^2^ statistic, which quantifies the proportion of total variability attributable to between-study differences [4]. Pooled estimates derived from the fixed-effect and random-effects meta-analyses were expressed as percentages and presented together with the associated 95% confidence intervals (CIs). The estimates were stratified by geographical region and by hospitalization status (when the information was available) defined as hospitalized only or studies in which the denominator was patients diagnosed in community/outpatient settings as well as inpatients. Similar meta-analyses were carried out for estimating the proportion of patients with the 3 leading complications of cerebral malaria, acute kidney injury (AKI), and pulmonary edema/acute respiratory distress syndrome (ARDS). Severe anemia was not included in the meta-analysis because our search strategy was not designed to capture the literature on vivax malaria and anemia comprehensively. Small study effect was assessed by using a linear regression test of funnel plot asymmetry and bias-adjusted estimates were reported using trim-and-fill method.

### Risk of bias assessment

The following aspects of the studies regarding patient selection and outcome assessment domains were considered: representativeness of the cohort (or cases in case–control studies), methodology used for ascertainment of exposure (microscopy, rapid diagnostic tests, and PCR), and if the assessment of exposure and outcome was blinded or not (laboratory procedures blinded or not). Case series and case reports were not included in the risk of bias assessment.

## Clinical features of vivax malaria

Studies of experimentally induced malaria and studies of returned United States servicemen provided early detailed descriptions of clinical features of vivax malaria in nonendemic areas. In a case series of 195 nonimmune adult prison volunteers given sporozoite-induced infections of the Chesson strain of *P*. *vivax* by US researchers in the 1940s, the most common symptoms reported, apart from fever, were headache, anorexia, nausea, myalgia, abdominal pain, eye pain, chest pain, cough, asthenia, and dizziness [5,6]. Vomiting, splenomegaly, epistaxis, urticaria, diarrhea, edema (not specified), jaundice, and hepatomegaly were typical clinical observations. A mean drop of hemoglobin (Hb) of 2.9 grams/dL was reported over the first 7 days of illness. Five participants who were observed over 15 days of illness experienced a mean Hb drop of 6.4 g/dL. By comparison, a systematic review of 1,975 patients of all ages in endemic areas with vivax malaria treated with chloroquine reported a maximum fall in mean Hb from 12.2 g/dL [95% CI 11.9, 12.5] before treatment to 11.6 g/dL [11.4, 11.9] 2 days later, with recovery by day 42 (12.9 g/dL [12.6, 13.2]) [7]. Patients with recurrent parasitemia had a Hb on average 0.7 g dL [0.5 to 0.9] lower on day 42 than patients with no recurrence (*p* < 0.001) [7]. *P*. *vivax* DNA has been detected by PCR from patients meeting the criteria for hyperreactive malarial splenomegaly; however, since *P*. *vivax* frequently cocirculates with *P*. *falciparum*, it has not been established definitively that *P*. *vivax* alone may be the cause [8].

In endemic regions, young children and pregnant women are particularly vulnerable to the negative clinical impacts of infection with *P*. *vivax*. There is no strong evidence for an interaction between vivax malaria and HIV, although few studies have looked at coinfection [9].

### Clinical impact of vivax malaria in young children

Symptoms of vivax malaria in children are nonspecific. Fever is typical but not universal and is often accompanied by rigors, vomiting, loose stools, poor feeding and irritability in infants, and sometimes, convulsions. Hepatosplenomegaly is common with an enlarged spleen described more frequently than an enlarged liver [10–14]. In areas where the population acquire immunity following repeated vivax infections, asymptomatic parasitemia is well recognized, and a high proportion of this is submicroscopic [13,15–17]. In a longitudinal study from a low-transmission area along the Thai–Myanmar border from 1991 to 1992, vivax malaria was most common in young children, and 43% of infections in children aged 4 to 15 years were asymptomatic, compared to only 16% of falciparum infections. Incidence of *P*. *vivax* declined with increasing age, while *P*. *falciparum* incidence peaked between 20 and 29 years [13]. A cohort study in 2006 of 264 children aged 1 to 3 years in a higher transmission setting in Papua New Guinea found that incidence of *P*. *vivax* decreased with age, while incidence of *P*. *falciparum* increased until 30 months of age and then remained stable. There was marked seasonal variation in *P*. *falciparum* infections, but not *P*. *vivax* infections, attributed to the propensity of *P*. *vivax* to relapse [18]. Relapse is common, and the periodicity depends on the infecting strain, the entomological inoculation rate, the number of sporozoites inoculated, the degree of immunity, and the treatment administered [19,20].

In Papua, Indonesia, young children experience a high number of recurrent episodes of vivax malaria early in life, and *P*. *vivax* in this area is highly chloroquine resistant. Observational data of 7,499 children below 5 years of age who presented to a large hospital between 2004 and 2013 and were diagnosed with vivax malaria showed that they were at greater risk of recurrent malaria than children with falciparum malaria [21]. Among 1,375 children with *P*. *vivax* infection admitted to hospital for parenteral antimalarial treatment, 28 (3.4%) died within 30 days. In the same analysis, the risk of dying within 30 days among children with *P*. *vivax* infection who were undernourished (weight for age or height/length for weight z scores below −3 SD of standard reference) was 15.5% (15/97). Children were followed up for 1 year, and the risk of dying between 31 and 365 days after their initial presentation in children diagnosed with vivax malaria was 1.0% (95% CI 0.8 to 1.3). The risk increased to 3.9% (3/77) in undernourished children. In another study from Venezuela, 26% of 78 children requiring hospitalization for vivax malaria were malnourished [22]. The relationship between malaria and nutritional status is complex and not well understood, with different studies suggesting that malnutrition may result from malaria, may increase susceptibility to malaria, or may be protective against malaria [21,23,24]. A serious consequence of repeated episodes of vivax malaria in children is severe anemia [25,26]. Another study from Papua, Indonesia found that infants aged 3 months or younger with vivax malaria were at higher risk of severe malarial anemia (Hb <5g/dL) than infants from the same population with falciparum malaria (odds ratio 2.4; 95% CI 1.0 to 5.9; *p* = 0.041) [12].

Malaria caused by *P*. *vivax* has been associated with cognitive impairment and poor school performance. In a study of children aged 6 to 14 years in Sri Lanka, who were monitored for 6 years, repeated episodes (≥3) of vivax malaria were associated with impaired performance in language and mathematics compared to children experiencing fewer than 3 episodes [27]. The same research team went on to conduct a double-blind, placebo-controlled trial of chloroquine prophylaxis in 587 children using attendance rates and language and mathematics scores to evaluate the intervention [28]. The children who received chloroquine experienced fewer episodes of malaria (55% reduction in incidence), were less likely to be absent from school, and scored higher in language and mathematics.

### Clinical impact of vivax malaria in pregnancy

Vivax malaria in pregnancy is associated with fetal loss, maternal anemia, low birth weight, and preterm birth with occasional reports of congenital infection [29,30]. Asymptomatic infection also occurs in pregnant women [31]. Much of the evidence for the impact of vivax malaria in pregnancy comes from large observational cohort studies of women receiving antenatal care in Asia and has accrued gradually [32,33]. A retrospective analysis of records of 17,613 pregnant women in the first trimester who received antenatal care in clinics along the Thailand–Myanmar border, where falciparum and vivax malaria were coendemic, between 1986 and 2010, found that the odds of miscarriage increased in women with asymptomatic (adjusted odds ratio 2.70, 95% CI 2.04 to 3.59) or symptomatic (3.99, 3.10 to 5.13) malaria with similar results for both *P*. *falciparum* and *P*. *vivax* [34].

A systematic review and meta-analysis examining the risk of stillbirth related to malaria in pregnancy, which included 59 studies, found that vivax malaria at the time of delivery, but not earlier during the pregnancy, increased the odds of stillbirth (2.8 [95% CI 0.8 to 10.2]) [35]. The relationship between vivax malaria and stillbirth was explored further in a retrospective analysis of a cohort of 61,836 women attending antenatal clinics along the Thailand–Myanmar border between 1986 and 2015, of whom 9,350 (15%) had malaria in pregnancy, and 526 (0.8%) had stillbirths [30]. The hazard of antepartum (and not intrapartum) stillbirth increased 2.2-fold (95% CI 1.1, 4.3; *p* = 0.021) among women who had symptomatic vivax malaria in the third trimester.

Data from the same cohort were used to assess the risk of preterm birth and small for gestational age (SGA) infants [36]. Having vivax malaria from 24 to 28 weeks’ gestation was associated with very preterm (≥28 and <32 weeks) birth (odds ratio 1.8 [95% CI 1.1, 2.9]), while between 28 and 32 weeks, it was associated with late preterm (≥32 and <37 weeks) birth (OR: 1.23 [1.0, 1.5]). Vivax malaria after 20 weeks’ gestation was associated with SGA infants (OR range: 1.12 to 1.54; *p*-value range: <0.001 to 0.138).

Between 2004 and 2006, a cross sectional study in Papua, Indonesia examined the relationship between malaria at delivery and pregnancy outcomes in 2601 women [37]. A decrease in birth weight associated with maternal *P*. *vivax* infection of 108 g (95% CI 17.5 to 199) was reported. *P*. *vivax* infection was also associated with an increased risk of moderate, but not severe, maternal anemia compared to no malaria with a reduction of Hb concentration of 0.4 g/dl (95% CI 0.1 to 0.7 g/dL).

A prospective observational study of 1,180 Brazilian pregnant women over 1 year from 2015 to 2016, 8% of whom had malaria (75% caused by *P*. *vivax*), showed that malaria episodes were associated with decreased birth weight and length and maternal anemia [38]. Birth weight z-scores showed a mean reduction of 0.35 (95% CI 0.14 to 0.57) in women with malaria in the second or third trimester compared to those with no malaria. The corresponding mean reduction in birth length z-scores was 0.31 (95% CI 0.08 to 0.54). Women with malaria in the second or third trimester also had a Hb concentration on average 0.33 g/100 mL lower (95% CI 0.05 to 0.62 g/100 mL). Of 637 cord blood samples tested by PCR, *P*. *vivax* was found in 4 cases.

Increased infant mortality has been associated with maternal vivax malaria during pregnancy. In a prospective study from a low-transmission setting along the Thailand–Myanmar border of a cohort of 1,495 mothers followed weekly throughout pregnancy and then with their infants until 1 year of life, malaria during pregnancy (any species) increased neonatal mortality by causing low birth weight, and fever in the week before delivery was also associated with increased infant mortality [39].

To date, interventional chemoprevention studies in pregnancy have not demonstrated a reduction in *P*. *vivax* malaria–related adverse pregnancy outcomes. In a randomized, double-blind, placebo-controlled trial of weekly chloroquine prophylaxis in 1,000 pregnant women on the Thai–Myanmar border, no women who received chloroquine had an episode of vivax malaria compared to 10% women in the placebo arm who experienced at least one [40]. Despite preventing episodes of vivax malaria, the proportions of women with anemia (hematocrit <30%) during the study in chloroquine and placebo arms were similar in both groups at 71.2% (336/472) and 71.4% (342/479), respectively (*p* = 0.76). No difference was observed between the groups in mean birth weight, proportion of low birth weight infants, or preterm delivery.

#### Placental changes with vivax malaria

Histopathological studies of placentas from women with vivax malaria during pregnancy have generally shown less evidence of intervillous inflammation or placental involvement in comparison to falciparum malaria [41–44]. In an ex vivo study reported from Thailand, *P*. *vivax* isolates were shown to adhere to chondroitin sulfate, the main receptor for adhesion of *P*. *falciparum* parasitized erythrocytes in the placenta [45].

### Severe vivax malaria

#### Results of rapid systematic review

**Fig 1:**
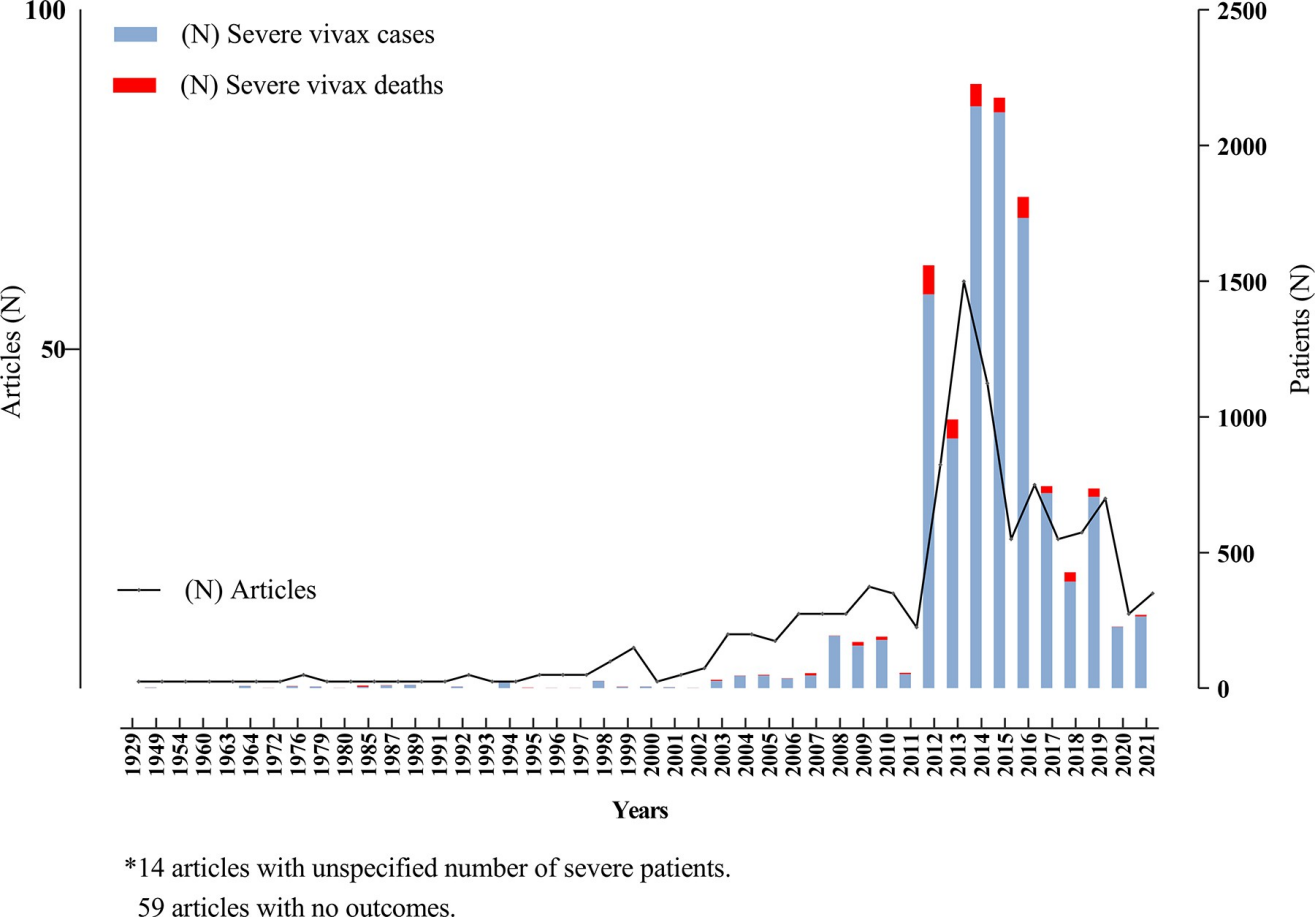
Summary of severe vivax patients, number of deaths reported, and articles by year (1928 to 2020).

**Fig 2:**
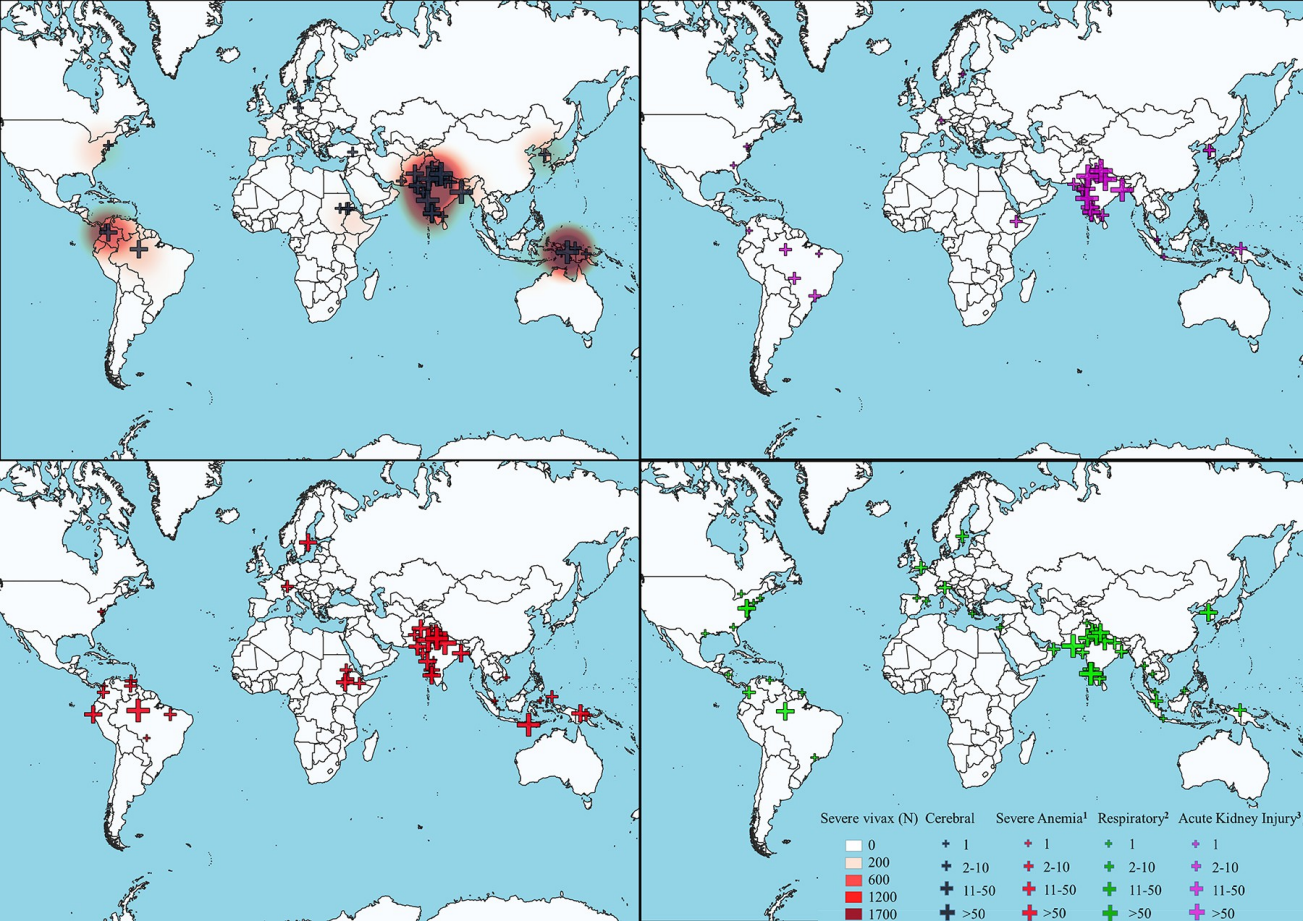
Number of severe vivax cases shown as a heat map with prevalence of severe manifestations using WHO definitions. ^1^Hb <5 g/dl for under 12 years old and <7 g/dl for above. ^2^Pulmonary edema defined by WHO (CXR confirmed or SPO2<92% + respiratory rate >30) or ARDS. ^3^Renal failure defined by WHO {plasma or serum creatinine >265 lM(3 mg/dl) or blood urea >20 mM}. Map links: https://qgis.org/en/site and https://thematicmapping.org/downloads/world_borders.php. The base layer of the map was downloaded from https://thematicmapping.org/downloads/world_borders.php. ARDS, acute respiratory distress syndrome; Hb, hemoglobin; WHO, World Health Organization.

The search for all severe manifestations of vivax malaria yielded 2,053 results. After screening title, abstract, and full text and removing duplicates, 1,696 articles were excluded (S1 Fig), leaving 423 for quantitative synthesis. There were 208 case reports or case series, 100 articles reporting prospective studies, and 115 retrospective studies. Fewer than 7 articles were published per year until 2002. Then the numbers gradually increased, peaking at 60 in 2013 (Fig 1). A total of 11 articles did not present the number of patients, only the number of severe complications; therefore, we cannot give the precise number of patients in our review. A total of at least 11,658 patients with at least 1 manifestation of severe vivax malaria were reported from 423 articles (Fig 2, S3 Table) across 45 countries, with over 60% of cases reported from 2 countries: India (43.9%, *n* = 5,114) and Indonesia (23.5%, *n* = 2,743). To confirm the diagnosis of *P*. *vivax* monoinfection, microscopy only was used in 35% (148) articles, molecular methods were used to confirm microscopy findings in 20% (85) articles, rapid diagnostic tests were used alone or in combination to confirm microscopy in 33% (139), PCR only was used in 4 cases, and diagnostic methods were not available in 8% (35) articles. There were only 5 cases with a parasite density reported in excess of 100,000/μL.

Laboratory diagnostics to exclude other comorbidities or concurrent illness, e.g., blood culture and dengue rapid diagnostic test, were performed to varying degrees of comprehensiveness in 224 (53%) articles and not done or not mentioned in 189 (44%).

#### Results of meta-analysis

A total of 92 studies were eligible for inclusion in the meta-analysis using both random effects and fixed effect models (S4 Table). Heterogeneity, assessed using the *I*^2^ statistic, was very high. From our risk of bias assessment focusing on 3 domains, representativeness of the population was at high or unclear risk of bias for 42% studies, half of the studies were retrospective and judged to be at high risk of bias and for ascertainment of exposure, which took diagnostic methods into account, while 72% were at moderate or unclear risk of bias (S5 Table). Most of the studies in the meta-analysis were from Asia (*n* = 67), and the majority (69) were conducted in hospital settings. In terms of patient numbers, of 4,547 patients with severe malaria included, approximately one-third were from Latin America. Only 85 studies could be assessed in the meta-analysis to estimate the proportion of severe vivax malaria because not all studies reported the number of patients with severe malaria, only the number of complications (Table 2). The random-effects estimate of the proportion of patients with WHO-defined severe disease was 0.7% [95% CI 0.19% to 2.57%] in studies that included all patients diagnosed with vivax malaria (inpatient and outpatient) and 7.11% [95% CI 4.30% to 11.55%] in studies of hospitalized patients (Table 2). Estimates using author definitions of severe malaria which included severe thrombocytopenia without bleeding were approximately 2-fold higher. Estimates adjusted for small study effects for all proportions are shown in the Supporting information (S6 Table). There were 4 studies exclusively in pregnant women (S7 Table). In a random-effects meta-analysis of these studies including 225 women, we estimated the proportion with severe malaria as 4.14% [95% CI 0.82% to 18.43%]. Further estimates of proportion with severe malaria stratified by hospitalization status within each region are presented as a Supporting information table (S8 Table).

**Table 2:** Estimating the proportion of vivax malaria that were classified as severe in studies eligible for inclusion in the meta-analysis.

			Estimates derived from meta-analysis of proportion		
Definition of severe malaria	Number of articles^a^	*n*/*N*	Fixed effect [95% CI]	*I* ^2^	Random effects [95% CI]
**WHO definition**					
Overall	84	4,518/818,808	0.55% [0.54% to 0.57%]	99.6%	4.37% [2.60% to 7.25%]
**Stratified by region**					
Africa	5	73/3,306	2.21% [1.76% to 2.77%]	93.7%	1.62% [0.18% to 12.94%]
Asia	66	3,572/330,131	1.08% [1.05% to 1.12%]	99.5%	7.29% [4.47% to 11.67%]
Oceania	1	16/1,946	0.82% [0.50% to 1.34%]	–	0.82% [0.50% to 1.34%]
South America	13	857/483,425	0.18% [0.17% to 0.19%]	99.2%	0.58% [0.12% to 2.87%]
**Stratified by settings**					
Hospitalized	66	2,594/35,822	7.24% [6.98% to 7.51%]	97.1%	7.11% [4.30% to 11.55%]
All (including outpatients)^b^	14	1,836/780,960	0.24% [0.22% to 0.25%]	99.5%	0.70% [0.19% to 2.57%]
Other^c^	4	88/2,026	4.34% [3.54% to 5.32%]	93.9%	1.92% [0.26% to 12.66%]
**All definitions**					
Overall	82	6,063/818,541	0.74% [0.72% to 0.76%]	99.7%	11.53% [8.09% to 16.18%]
**Stratified by region** ^d^					
Africa	5	202/3,306	6.11% [5.34% to 6.98%]	97.9%	9.69% [3.54% to 23.84%]
Asia	64	4,226/329,864	1.28% [1.24% to 1.32%]	99.6%	16.02% [11.46% to 21.94%]
Oceania	1	100/1,946	5.14% [4.24% to 6.21%]	–	5.14% [4.24% to 6.21%]
South America	13	1,535/483,425	0.32% [0.30% to 0.33%]	99.5%	2.24% [0.72% to 6.78%]
**Stratified by settings** ^d^					
Hospitalized	65	3,420/3,5742	9.57% [9.27% to 9.80%]	97.5%	17.74% [13.19% to 23.42%]
All (including outpatients)^b^	14	2,560/780,960	0.33% [0.32% to 0.34%]	99.6%	1.57% [0.61% to 4.00%]
Other^c^	3	83/1,839	4.51% [3.65% to 5.56%]	96.8%	5.06% [1.18% to 19.22%]

*n* = Number of patients with severe vivax malaria; *N* = total number of patients with vivax malaria.

^a^Studies that were carried out exclusively among pregnant women are excluded; studies that include few or some pregnant women were not excluded.

^b^Studies that predominantly reported data on outpatients settings were also included.

^c^Other includes studies that did not mention the settings and the studies in which the number of patients who were hospitalized or treated outpatients could not be reliably extracted.

^d^Estimates stratified by settings within each region is presented in additional file (S8 Table).

When region and settings were adjusted using a multivariable logistic regression model with study as the random effects, the difference between the settings (hospitalization versus not) remained, whereas the difference between regions (Africa versus Asia and South America versus Africa) was not significant. Interaction between region and settings was not significant (*p =* 0.278 for the test of interaction using all definitions of severe vivax and *p =* 0.081 for WHO definition).

CI, confidence interval; WHO, World Health Organization.

### Cerebral malaria

We found 163 articles describing 1,502 patients with cerebral manifestations of vivax malaria. Among the cerebral complications in these patients, 645 (42.9%) fulfilled WHO criteria for cerebral malaria (S3 Table), 278 (18.5%) had seizures only with no other features of cerebral malaria), and 579 (38.6%) had cerebral symptoms such as altered consciousness or sensorium, confusion, and/or low but unquantified Glasgow Coma Scores. Over 75% of cerebral malaria cases were reported from one of India 53.7% (807/1,502), Colombia 11.9% (178/1,502), or Indonesia 10.2% (153/1,502) (Fig 2). A small number of articles described retinal changes, including hemorrhages, cotton wool spots, and papilledema occurring in complicated vivax malaria [46], Vivax malaria has also been reported as presenting as a cerebral infarct or cerebral venous sinus thrombosis [47,48].

From these reports, 90 articles were eligible for inclusion in the meta-analysis of cerebral vivax malaria. The random-effects estimate of the proportion of the patients from all studies with WHO-defined cerebral malaria was 0.62% [95% CI 0.33% to 1.18%] among hospitalized patients and 0.02% [95% CI 0.00% to 0.13%] among patients from studies that included all patients diagnosed with vivax malaria (inpatient and outpatient) (Table 3).

**Table 3:** Estimating proportion of patients with cerebral malaria, renal complications, and respiratory complications in studies eligible for inclusion in the meta-analysis.

			Estimates derived from meta-analysis of proportion		
	Number of articles^a^	*n*/*N*	Fixed effect [95% CI]	*I* ^2^	Random effects [95% CI]
**Cerebral malaria**					
**WHO definition**					
Overall	90	636/820,671	0.08% [0.07% to 0.08%]	97.7%	0.28% [0.14% to 0.56%]
Hospitalized	71	466/37,602	1.24% [1.13% to 1.36%]	89.8%	0.62% [0.33% to 1.18%]
All (including outpatients)^b^	14	156/780,960	0.02% [0.02% to 0.02%]	98.3%	0.02% [0.00% to 0.13%]
Other^c^	5	14/2,109	0.66% [0.39% to 1.12%]	80.9%	0.10% [0.00% to 3.68%]
**All definitions**					
Overall	90	962/820,671	0.12% [0.11% to 0.12%]	98.3%	0.83% [0.51% to 1.35%]
Hospitalized	71	631/37,602	1.68% [1.55% to 1.81%]	91.2%	1.43% [0.90% to 2.25%]
All (including outpatients)^b^	14	310/780,960	0.04% [0.04% to 0.04%]	98.5%	0.10% [0.03% to 0.32%]
Other^c^	5	21/2,109	1.00% [0.65% to 1.52%]	84.1%	0.87% [0.13% to 5.69%]
**Renal**					
**WHO definition**					
Overall	89	447/820,351	0.05% [0.05% to 0.06%]	89.7%	0.18% [0.08% to 0.39%]
Hospitalized	70	405/37,282	1.09% [0.99% to 1.2%]	87.7%	0.51% [0.26% to 0.98%]
All (including outpatients)^b^	14	39/780,960	0.00% [0.00% to 0.01%]	91.2%	0.01% [0.00% to 0.10%]
Other^c^	5	3/2,109	0.14% [0.05% to 0.44%]	0.0%	0.14% [0.05% to 0.44%]
**All definitions**					
Overall	88	810/820,248	0.1% [0.09% to 0.11%]	95.0%	0.49% [0.25% to 0.97%]
Hospitalized	69	759/37,179	2.04% [1.9% to 2.19%]	94.1%	1.29% [0.73% to 2.26%]
All (including outpatients)^b^	14	48/780,960	0.01% [0.00% to 0.01%]	93.0%	0.02% [0.00% to 0.19%]
Other^c^	5	3/2,109	0.14% [0.05% to 0.44%]	0.0%	0.14% [0.05% to 0.44%]
**Respiratory**					
**WHO definition**					
Overall	87	414/819,669	0.05% [0.05% to 0.06%]	92.6%	0.23% [0.11% to 0.47%]
Hospitalized	69	352/37,233	0.95% [0.85% to 1.05%]	86.6%	0.66% [0.38% to 1.13%]
All (including outpatients)^b^	13	57/780,327	0.01% [0.01% to 0.01%]	85.5%	0.00% [0.00% to 0.07%]
Other^c^	5	5/2,109	0.24% [0.1% to 0.57%]	55.0%	0.27% [0.06% to 1.16%]
**All definitions**					
Overall	87	848/819,669	0.10% [0.10% to 0.11%]	98.1%	0.66% [0.41% to 1.05%]
Hospitalized	69	450/37,233	1.21% [1.10% to 1.32%]	90.4%	1.00% [0.62% to 1.61%]
All (including outpatients)^b^	13	393/780,327	0.05% [0.05% to 0.06%]	98.8%	0.18% [0.06% to 0.53%]
Other^c^	5	5/2,109	0.24% [0.10% to 0.57%]	55.0%	0.27% [0.06% to 1.16%]

*n* = Number of patients with the given complications; *N* = total number of patients with vivax malaria.

^a^Studies that were carried out exclusively among pregnant women are excluded; studies that include few or some pregnant women were not excluded.

^b^Studies that predominantly reported data on outpatients settings were also included.

^c^Other includes studies that did not mention the settings and the studies in which the number of patients who were hospitalized or treated outpatients could not be reliably extracted.

CI, confidence interval; WHO, World Health Organization.

### Postmalaria neurological syndrome (PMNS)

We found 8 (0.07%) reports of PMNS: 4 cases of acute disseminated encephalomyelitis (ADEM), 2 of Guillain–Barré syndrome, 1 report of a patient with behavior change, and 1 of myelitis.

### Respiratory manifestations of severe vivax malaria

Of 1,202 patients with respiratory complications reported from 97 articles, 258 (21.5%) fulfilled WHO criteria for pulmonary edema (chest radiograph confirmed or O_2_ saturations <92% with respiratory rate more than 30/min); 330 (26.6%) were reported to have ARDS; and 593 (48.6%) had other respiratory complications such as respiratory distress (not otherwise specified), pleural effusion, and pneumothorax (Fig 2).

From the meta-analysis of respiratory complications in 87 studies (Table 3), the proportion of patients with WHO-defined pulmonary edema was 0.66% [95% CI 0.38% to 1.13%] in studies among patients who were hospitalized and 0.00% [95% CI 0.00% to 0.07%] in studies that included all patients diagnosed with vivax malaria (inpatient and outpatient). Only a few articles (32/138) enumerated the time of onset of pulmonary symptoms relative to starting malaria treatment; symptoms developed before malaria therapy in 96 patients, after in 84 patients, and for the remainder the time of onset were not specified.

#### Lung histology

Histological findings from one fatal case report from India included alveolar damage and hyaline membrane formation, typical of ARDS [49]. A Brazilian postmortem series of 13 patients found ARDS and pulmonary edema in 2 and 5 cases, respectively. The pathologist reported scattered parasitized red blood cells in capillaries in the lungs of one of the patients with ARDS.

### Renal manifestations of severe vivax malaria

Among 1,694 patients with biochemical evidence of impaired renal function, 915 (54.0%) met WHO criteria for AKI (S3 Table), 233 (13.8%) cases were classified using the Kidney Disease: Improving Global Outcomes (KDIGO) [50] or Risk, Injury, Failure; Loss, End-Stage Renal Disease (RIFLE) [51] classifications, and 546 (32.2%) were categorized using local definitions with different urea or creatinine cutoffs. More than 75% (1,694/1,272) of renal complications were reported from India (Fig 2). From the random-effects meta-analysis of renal complications from 89 eligible articles, we estimated that 0.51% [95% CI 0.26% to 0.98%] patients met WHO criteria for AKI in studies of hospitalized patients and 0.01% [95% CI 0.00% to 0.10%] in studies that included all patients diagnosed with vivax malaria (inpatient and outpatient) (Table 3).

#### Renal histology

We found reports of renal biopsy results from 45 patients with renal impairment and vivax malaria (S9 Table). The most common histological diagnosis was acute tubular necrosis (*n* = 20), with a second diagnosis reported in 9 cases; cortical necrosis was reported in 16, thrombotic microangiopathy in 12, and mesangiocapillary glomerulonephritis in 7 patients.

## Mortality from vivax malaria

A total of 553 deaths were associated with severe vivax malaria (S3 Table). Out of these, 334 were from 75 studies eligible for inclusion in the random-effects meta-analysis, and the overall estimate of mortality (all studies, regardless of setting) was 0.27% [0.15% to 0.50%] (Table 4). There were no reports of deaths from the African region.

**Table 4:** Estimating mortality in studies eligible for inclusion in the meta-analysis.

			Estimates derived from meta-analysis of proportion		
	Number of articles^a^	*n*/*N*	Fixed effect [95% CI]	*I* ^2^	Random effects [95% CI]
Overall	75	334/814,505	0.04% [0.04% to 0.05%]	96.2%	0.27% [0.15% to 0.5%]
**Stratified by region**					
Africa	2	0/2,263	0.00% [0.00% to 0.17%]^d^	to	0.00% [0.00% to 0.17%]^d^
Asia	62	271/329,257	0.08% [0.07% to 0.09%]	93.8%	0.50% [0.29% to 0.84%]
Oceania	–	–	–	–	–
South America	12	63/482,985	0.01% [0.01% to 0.02%]	95.8%	0.03% [0.00% to 0.24%]
**Stratified by settings**					
Hospitalized	61	234/36,087	0.65% [0.57% to 0.74%]	80.6%	0.56% [0.35% to 0.92%]
All (including outpatients)^b^	11	82/778,041	0.01% [0.01% to 0.01%]	95.2%	0.01% [0.00% to 0.07%]
Other^c^	3	18/377	4.77% [3.03% to 7.45%]	75.6%	2.50% [0.59% to 9.98%]

*n* = Number of patients who died; *N* = total number of patients with vivax malaria.

^a^Studies that were carried out exclusively among pregnant women are excluded; studies that include few or some pregnant women were not excluded.

^b^Studies that predominantly reported data on outpatients settings were also included.

^c^Other includes studies that did not mention the settings and the studies in which the number of patients who were hospitalized or treated outpatients could not be reliably extracted.

^d^95% CIs were obtained using Wilson’s method ignoring the site effects.

CI, confidence interval.

### Postmortem evidence of severe malaria

In a retrospective review of the postmortem findings of 17 patients who had died with vivax malaria between 1996 and 2010 in a single centre in Brazil, it was concluded that vivax malaria was the probable cause of death or contributed to the fatal outcome in 13 cases aged between 8 and 88 years [52]. Pulmonary edema or ARDS were the leading causes of death (*n* = 7), coexisting with splenic rupture in 2 patients. The other causes of death were multiple organ dysfunction (*n* = 3, including one with a cerebrovascular accident), primaquine-induced hemolytic anemia in the context of glucose-6-phosphate dehydrogenase (G6PD) deficiency (*n* = 2), and isolated splenic rupture (*n* = 1). A total of 7 patients had comorbidities such as hepatic cirrhosis or pulmonary emphysema. Postmortem findings consistent with ARDS were reported from one fatal case in India [49].

Postmortem histology from one fatal case of “infection with (the) large tertian parasite” in 1902 [1] found a spleen containing numerous large parasites, tubular degeneration in the kidneys, pigment in lung capillaries but no parasites, and very little evidence of pigment or parasitized erythrocytes in the brain. Another postmortem study of a Vietnamese patient with severe vivax malaria who died suddenly but was fully conscious on presentation found no evidence of sequestration of parasitized erythrocytes in the cerebral microvasculature [53].

## Pathophysiology of severe vivax malaria

Theories as to the pathophysiological processes underlying severe disease include immune dysfunction [54–59], parasite strain–specific virulence [60–62], inflammation triggered by cytokines, and endothelial cell dysfunction [63]. A proteomic study testing serum from Indian patients with vivax malaria of differing levels of severity as well as controls has given signals of possible involvement of oxidative stress pathways, cytoskeletal regulation, lipid metabolism, and complement cascades [64]. Waning immunity as transmission goes down and increasing resistance of *P*. *vivax* to chloroquine have been put forward as possible explanations for increasing reports of severe vivax malaria [54,65]. Increased expression of *pvcrt-o* (*P*. *vivax* chloroquine resistance transporter-o), *pvmdr-1* (*P*. *vivax* multidrug resistance gene-1), and *vir* (variant interspersed repeats) genes has been shown in small numbers of patients with severe compared to uncomplicated vivax malaria [66–68].

Studies of patients with malaria have shown *P*. *vivax* evokes relatively higher concentrations of pro- and anti-inflammatory cytokines, such as tumor necrosis factor (TNF), and other markers of host immune response than *P*. *falciparum* at similar parasite densities [69,70]. While these findings can explain the lower pyrogenic parasite densities of vivax infections compared to falciparum, TNF is not thought to play a causal role in coma and cerebral manifestations of severe falciparum malaria [71]. Some in vitro studies have demonstrated some cytoadhesive properties of *P*. *vivax* parasitized erythrocytes [72]; however, cytoadherence and/or sequestration have not been demonstrated in vivo, and there are no published postmortem studies of patients diagnosed with cerebral vivax malaria. Binding of uninfected erythrocytes to parasitized erythrocytes (rosetting) does occur, which may lead to impaired circulation [73,74]. Modest (2-fold) increases in peripheral parasite densities in severe disease are accompanied by larger (7-fold) increases in circulating parasite lactate dehydrogenase, suggesting the possibility of parasitized erythrocytes accumulating elsewhere [75].

Impairment of endothelial function has been shown in Malaysian patients with vivax malaria [63]. Endothelial injury and excessive inflammation could also explain ARDS presentations. In a study of pulmonary gas transfer in Indonesian adults with falciparum or vivax malaria diffusing capacity for carbon monoxide (DLCO) in patients with vivax malaria was 93% (95% CI 87 to 104) of predicted values at baseline and declined for 2 weeks after treatment to 84% (95% CI 72 to 95) [76]. Evidence for cytoadherence in the pulmonary microvasculature is not that compelling, although scattered parasitized red blood cells in pulmonary capillaries have been described in one patient postmortem, and *P*. *vivax* has been shown to adhere to human lung microvascular endothelial cells (HMVEC-L) in vivo, albeit to a lesser degree than *P*. *falciparum* [52,77].

Increased concentrations of platelet-associated IgG (PAIgG) have been reported in patients with thrombocytopenia and vivax malaria, of unclear significance [78]. It has also been postulated that increased levels of macrophage colony–stimulating factor in vivax and falciparum malaria may result in increased macrophage-mediated platelet destruction and thrombocytopenia [79].

The anemia associated with *P*. *vivax* malaria has been reviewed in detail by Douglas and colleagues [25].

Mechanisms by which malaria leads to anemia include destruction of parasitized erythrocytes, destruction of nonparasitized erythrocytes, and dyserythropoeisis. Unlike in falciparum malaria, it is unusual to have parasite densities more than 2% infected erythrocytes in acute vivax malaria [80]. *P*. *vivax* has a predilection for invading younger erythrocytes, which has been thought to be one reason why unrestricted parasite multiplication does not occur as for *P*. *falciparum*. However, severity of anemia may be comparable, or worse. Collins and colleagues postulated that destruction of reticulocytes as they are produced could account for this [81]. However, Kitchen observed in another study published in 1938 that, even when reticulocyte numbers increase, only a fixed proportion will be infected; therefore, other factors may be more important [82]. Survival of uninfected red cells is shortened after an episode of malaria [81]. A mathematical modeling study using parasitemia and Hb data from patients receiving *P*. *falciparum* malariatherapy for neurosyphilis estimated that 8.5 nonparasitized erythrocytes were destroyed for each parasitized erythrocyte [83]. In malaria caused by *P*. vivax, the number may be even higher, given that parasites densities are lower. However, there is only indirect evidence for this. In a study of mean erythrocyte survival after *P*. *falciparum* or *P*. *vivax* parasitemia in 35 Thai patients who received transfusions of either labeled autologous or donor erythrocytes once their parasitemia had cleared, a similar reduction in erythrocyte survival was seen, regardless of the infecting species and did not appear to be antibody or complement mediated [84]. In a Colombian study comparing patients with uncomplicated and complicated (defined as platelets <50,000/μL, abnormal liver enzymes, or hypoglycemia) vivax malaria, there was an association between autoimmune antibodies and Hb concentrations in complicated disease. There was also a correlation with levels of atypical memory B cells, which have been hypothesized to contribute to anemia in malaria by secreting antibodies against phosphatidylserine on uninfected erythrocytes [85].

In malaria endemic areas, the degree of anemia associated with *P*. *vivax* infections depends on transmission intensity, patient age, and by extension degree of acquired immunity, strain relapse periodicity, and choice of antimalarial treatment, e.g., more slowly eliminated drugs will suppress the first relapse, allowing time for hematological recovery, while treatment failure due to resistance will impede recovery.

## Discussion

The global burden of vivax infections is vast, with estimates ranging from 7 to 14 million cases annually, leading to a substantial clinical impact, particularly in young children and pregnant women [86,15]. While it is now generally accepted that vivax malaria may manifest as severe disease, uncertainties remain. Reports of severe vivax malaria have increased over the last decade, but it is unclear to what extent this represents a genuine increase in case numbers, increased recognition of the disease, or misattribution in patients with other diagnoses and incidental parasitemia. In Manaus, Brazil, where a consistent case definition has been used over time, the incidence of severe disease has been increasing [87]. Increasing chloroquine resistance might be expected to lead to more severe disease, although in Papua, Indonesia, switching from chloroquine to artemisinin combination therapy did not lead to a mortality reduction in patients hospitalized with vivax malaria, suggesting that relapse prevention may be more important to reduce deaths [88]. This is supported by another analysis of more than 20,000 patients with vivax malaria in the same location showing that *P*. *vivax* infection was a risk factor for representation to hospital and contributed to increased mortality [89].

There are major disparities in frequencies of reporting certain complications of vivax malaria from different parts of the world (Fig 2) and a near absence of reporting of severe vivax malaria from some Southeast Asian countries, e.g., Thailand, Myanmar, Vietnam, and Lao PDR. Strain-specific virulence has been described in both induced malaria studies [90] and natural infections such as in the former United Soviet Socialist Republic where outbreaks of “fulminant malaria” caused by *P*. *vivax* were well described in the 1940s [91]. While this may be the explanation for the heterogeneous distribution of severe disease, the fact that asymptomatic vivax parasitemia is common means that a degree of misattribution is inevitable. This has been shown in individual fatal cases for whom exhaustive attempts were made to rule out other causes [52,92]. It is also suggested by variable histological findings from patients with vivax malaria who have had a renal biopsy. Only about half of the studies included in this review described attempts to exclude other diagnoses, e.g., sepsis. A 1 year prospective study from Kolkata examined the rate of concomitant bacteremia with *P*. *vivax* parasitemia and found that 6/89 (6.7% [95% CI 3.1% to 13.9%]) of patients with *P*. *vivax* infection were bacteremic [93].

Our estimate of mortality resulting from *P*. *vivax* infection from the random-effects meta-analysis ranged from 0.01% [0.00% to 0.07%] (studies of all patients, i.e., outpatient and inpatient cases) to 0.56% [0.35% to 0.92%] in studies of hospitalized patients only. These results were similar to a case fatality rate estimate of 0.3% (353/46,411) from a fixed-effect meta-analysis in another systematic review, which used a modified WHO definition of severe malaria and included thrombocytopenia [94]. However, these estimates should be interpreted with caution given the risk of bias assessment of included studies.

Detailed studies from a small number of research groups so far have not identified a common pathophysiological process underlying different complications of vivax malaria. Sequestration of parasitized red blood cells in the microcirculation, which is the pathophysiological hallmark of severe falciparum malaria, has been not been demonstrated.

ARDS has been reported with infection from all *Plasmodium* species causing malaria in humans. Incidence rates of ARDS in severe falciparum malaria have been shown to vary between 2% and 25% and are more likely to lead to a fatal outcome than ARDS with *P*. *vivax* [95,96].

The need for an adapted case definition for severe vivax malaria has been recognized for over a decade. Reporting by different groups is inconsistent, impeding gathering reliable incidence data or conducting research. A Brazilian study looking at predictors of intensive care unit (ICU) admission in patients with vivax malaria found that many of the criteria in the severe falciparum malaria definition were predictive, with the exception of hyperbilirubinemia [87]. The importance of thrombocytopenia as a diagnostic criterion for severe vivax malaria has been debated [97]. Lampah and colleagues from Papua, Indonesia reported the mortality risk of *P*. *vivax* infections in patients presenting to a referral hospital with severe thrombocytopenia to be 1.5% (25/1650) below 50 × 10^9^/L and 3.6% (6/168) below 20 × 10^9^/L in patients with *P*. *vivax* and proposed the latter threshold as a severity criterion [98]. The utility of this in routine practice would need to be demonstrated since complete blood count is not available in many outpatient settings where malaria is diagnosed and treated [98]. Conversely, incorporating platelet counts into case definitions of severe falciparum malaria using >200,000 per μL to rule out severe malaria has been proposed as a means to improve the specificity of clinical and parasitological diagnosis in a mathematical modeling study. Results suggested that one-third of 2,220 Kenyan children included in studies had been misdiagnosed as having severe malaria [99]. Very few articles reported platelet counts associated with severe syndromes in our review so we were unable to explore this.

Limitations of our rapid systematic review include limiting our search to the English language and omitting to search other databases such as the Literatura Latino-Americana e do Caribe em Ciências da Saúde (LILACS) database. A systematic review of the Brazilian published and gray literature in 2012 described a similar array of complications from *P*. *vivax* infection to those reported here [100]. The literature is dominated by case series and reports and by hospital-based studies increasing the risk of bias toward more severely ill patients. Our formal risk of bias assessment of studies included in the meta-analysis indicated a moderate to high risk of bias. There was also evidence of small study effects which was shown in an earlier systematic review by Naing [101].

## Conclusions

Vivax malaria has emerged from the shadow of falciparum malaria over the last decade with improved recognition of the negative clinical impacts associated with this relapsing infection.

Preventing severe anemia associated with relapsing vivax malaria, particularly in very young children, is a priority to reduce morbidity and mortality in this group. Progress has been slowed by low uptake of antihypnozoiticidal treatment with 8-aminoquinoline drugs due to fears of hemolysis in patients with G6PD deficiency, compounded by lack of access to G6PD testing [102].

Prevention of infection with *P*. *vivax* in pregnancy may need to target young women preconception in order to prevent the risk of relapse during pregnancy and the consequent negative impacts of maternal anemia, increased fetal loss, and low birth weight [103]. Severe clinical presentations of vivax malaria are now well recognized, although knowledge gaps persist in terms of understanding the underlying pathophysiology of different complications and the apparent heterogeneity in incidence worldwide. An adapted case definition of severe vivax malaria would facilitate surveillance and future research.

## Supporting information

S1 Fig PRISMA flow diagram. PRISMA, Preferred Reporting Items for Systematic Reviews and Meta-Analyses.
(PDF)

S1 Table Search keywords and results. (DOCX)

S2 Table PRISMA checklist. PRISMA, Preferred Reporting Items for Systematic Reviews and Meta-Analyses.
(DOCX)

S3 Table Summary of severe manifestations of vivax malaria. (DOCX)

S4 Table List of studies included in meta-analyses. (XLSX)

S5 Table Risk of bias assessment of studies in meta-analyses. (XLSX)

S6 Table Assessment of small study effects. (DOCX)

S7 Table Summary of studies of severe vivax malaria in pregnant women. (DOCX)

S8 Table Proportion of severe vivax stratified by settings within each region. (DOCX)

S9 Table Summary of renal biopsy histology from patients with vivax malaria. (DOCX)

## Abbreviations

ADEM: acute disseminated encephalomyelitis
AKI: acute kidney injury
ARDS: acute respiratory distress syndrome
CI: confidence interval
DLCO: diffusing capacity for carbon monoxide
G6PD: glucose-6-phosphate dehydrogenase
Hb: hemoglobin
HMVEC-L: human lung microvascular endothelial cell
ICU: intensive care unit
KDIGO: Kidney Disease: Improving Global Outcomes
LILACS: Literatura Latino-Americana e do Caribe em Ciências da Saúde
PAIgG: platelet-associated IgG
PMNS: postmalaria neurological syndrome
PRISMA: Preferred Reporting Items for Systematic Reviews and Meta-Analyses
RIFLE: Risk, Injury, Failure
Loss: End-Stage renal disease
SGA: small for gestational age
TNF: tumor necrosis factor
WHO: World Health Organization
