# Supplementary material for: Clinical impact of vivax malaria: A collection review
Source: PLoS Med. 2022 Jan 18;19(1):e1003890. doi: 10.1371/journal.pmed.1003890 (PMC8765657; doi:10.1371/journal.pmed.1003890)
Supplement: S1 Fig — PRISMA, Preferred Reporting Items for Systematic Reviews and Meta-Analyses. (PDF) [file pmed.1003890.s001.pdf]

Figure S1. PRISMA flow chart for severe vivax malaria review

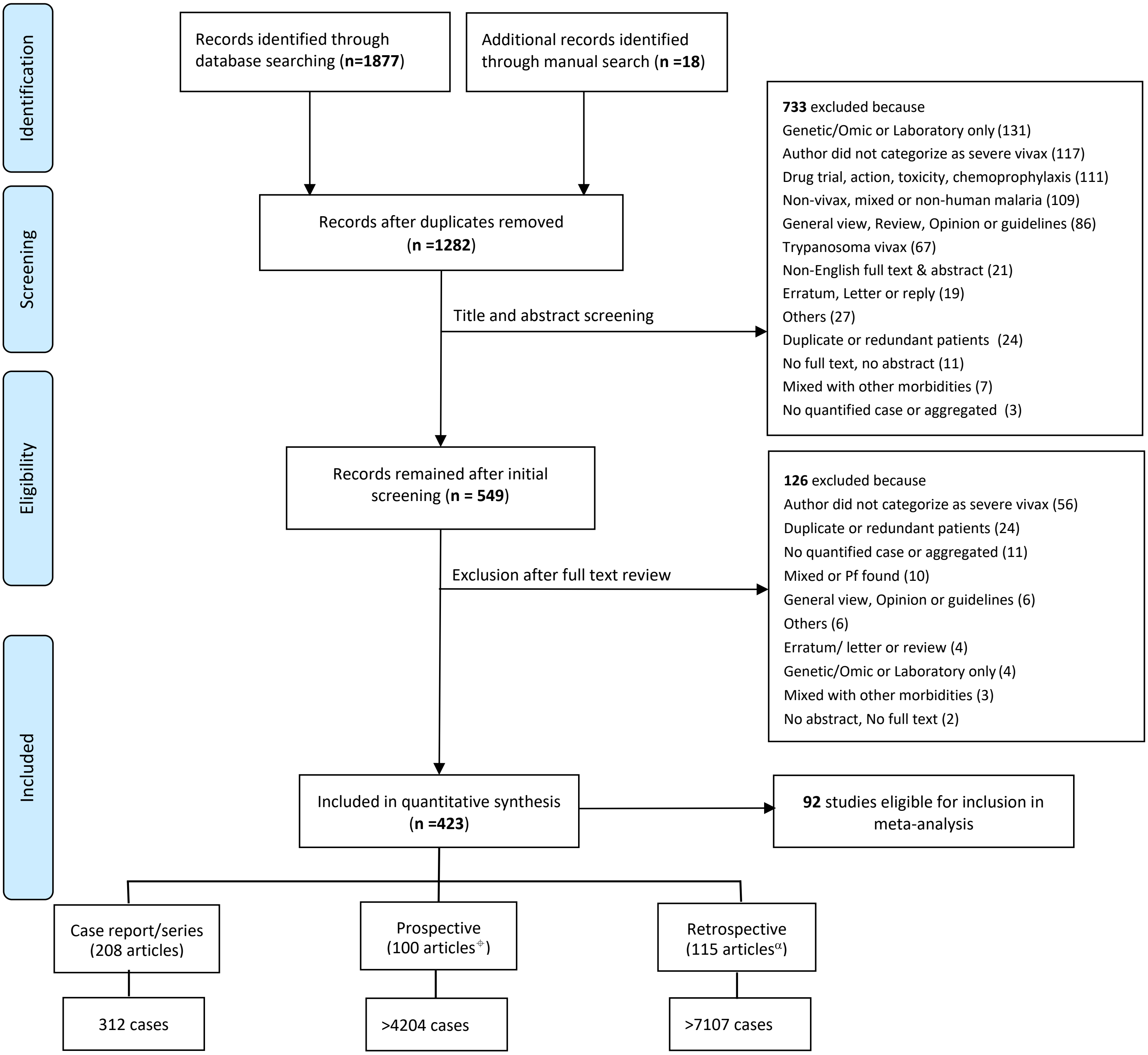

3[⌖], 8[α] with unquantified number of severe patients (complications only)
