# Supplementary material for: Clinical impact of vivax malaria: A collection review
Source: PLoS Med. 2022 Jan 18;19(1):e1003890. doi: 10.1371/journal.pmed.1003890 (PMC8765657; doi:10.1371/journal.pmed.1003890)
Supplement: S1 Table — (DOCX) [file pmed.1003890.s002.docx]

**S1 Table. Search keywords and results**

| Search keywords | Pubmed |
| --- | --- |
| ((vivax[Title/Abstract]) AND (severe[Title/Abstract])) | 978 |
| (("vivax"[Title]) AND ("fatal"[Title])) OR (("vivax"[Title]) AND ("death"[Title])) | 12 |
| ("vivax"[Title]) AND ("cerebral"[Title/Abstract]) | 75 |
| ("vivax"[Title/Abstract]) AND ("seizure*" OR "convulsion*"[Title/Abstract]) | 57 |
| ("vivax"[Title/Abstract]) AND ("hepatic"[Title/Abstract]) | 111 |
| ("vivax"[Title/Abstract]) AND ("renal" OR "kidney*"[Title/Abstract]) | 215 |
| "vivax"[Title] AND ("severe anaemia"[Title/Abstract] OR "severe anemia"[Title/Abstract]) | 77 |
| ("vivax"[Title]) AND ("disseminated intravascular coagulation"[Title/Abstract]) | 13 |
| ("vivax"[Title/Abstract]) AND ("cardi*" [Title/Abstract]) | 51 |
| ("vivax"[Title/Abstract]) AND ("acido*"[Title/Abstract]) | 34 |
| ("vivax"[Title/Abstract]) AND ("ARDS" OR "respirat*"[Title/Abstract]) | 172 |
| ("vivax"[Title]) AND ("thrombocytop*"[Title]) | 53 |
| ("vivax"[Title]) AND ("bleeding"[Title/Abstract]) | 29 |
| Total | 1877 |
| Duplicate | 613 |
| N after de-duplication | 1264 |
| Additional records identified through manual search and from other systematic reviews | 18 |
| Records at screening | 1282 |
