# Supplementary material for: Clinical impact of vivax malaria: A collection review
Source: PLoS Med. 2022 Jan 18;19(1):e1003890. doi: 10.1371/journal.pmed.1003890 (PMC8765657; doi:10.1371/journal.pmed.1003890)
Supplement: S3 Table — (DOCX) [file pmed.1003890.s004.docx]

**S3 Table. Summary of severe manifestations of vivax malaria from all included articles**

| Severe manifestation (% of total severe N) | n (%) | n (India) | Died^1^ | Survived | Unknown |
| --- | --- | --- | --- | --- | --- |
| Cerebral (12.9%)^2^ | **1502** | **807** | 121 (8.8) | 662 (44.7) | 719 (46.5) |
| Cerebral (GCS<11 or Author said WHO) | 645 | 450 |  |  |  |
| Seizure (Standalone) | 278  () | 171  171 |  |  |  |
| Cerebral (Other) | 579 | 186  1861272 |  |  |  |
| Renal (14.5%) | **1694** | **1272** | 137 (8.1) | 1046 (61.7) | 511 (30.2) |
| AKI (WHO) (Cre >3 mg/dl or BUN>20 mM) | 915 | 819 |  |  |  |
| AKI (RIFLE-IFLE or KDIGO) | 233 | 334 |  |  |  |
| AKI (Other) | 546 | 119 |  |  |  |
| Respiratory (10.3%)^3^ | **1202** | **478** | 144 (12.0) | 476 (39.6) | 582 (48.4) |
| Pulm. oedema (WHO)(CXR Or O2<92% RR>30) | 258 | 120 |  |  |  |
| ARDS | 351 | 115 |  |  |  |
| Other respiratory manifestations | 593 | 243 |  |  |  |
| Severe anaemia (22.3%) | **2594** | **875** |  |  |  |
| Per WHO (Hb<5 if <12 years or <7 for adult) | 2032 | 690 | 24 (1.2) | 960 (47.2) | 1048 (51.6) |
| Other Hb cut-offs | 562 | 185^†^ |  |  |  |
| Jaundice (16.8%) | **1952** | **1418** | 93 (4.8) | 1327 (67.9) | 532 (27.3) |
| Jaundice (WHO) Tbil>3 mg/dl | 1348 | 1021 |  |  |  |
| Jaundice (Other) | 604 | 397 |  |  |  |
| Shock (6.6%) | **774** | 58 | 57 (7.3) | 475 (61.4) | 242 (31.3) |
| DIC (1.8%) | **208** | 147 | 16 (7.7) | 130 (62.5) | 62 (29.8) |
| Acidosis (2.7%) | **317** | 246 | 35 (11.0) | 239 (75.4) | 43 (13.6) |
| PMNS, ADEM, Guillain-Barré (0.07%) | **8** |  |  |  |  |
| Splenic rupture/abscess/hematoma (0.6%) | **68** | 9^††^ |  |  |  |
| MODS (1.4%) | **158** | 115 |  |  |  |
| Hypoglycemia (1.1%) | **129** | 58 |  |  |  |
| Hepatic dysfunction^4^ (0.9%) | **109** | 61 |  |  |  |
| Other^5^ (1.9%) | **220** | 97 |  |  |  |
| Unspecified severe^6^ (3.6%) | **414** | 168 |  |  |  |
| Thrombocytopenia: any cut-off (39.5%) | **4596** | **1753** |  |  |  |
| Platelet count < 20, 000/µL | 715* | 375 |  |  |  |
| Platelet count < 50, 000/µL | 2505** | 449 |  |  |  |
| Platelet count < 150, 000/ µL | 1376 | 929 |  |  |  |
|  |  |  |  |  |  |
| Total N of deaths (%)^7^ | **553 (0.05%)** | **334** |  |  |  |
| Total patients with severe manifestations^8^ | **11,658** | **5114** |  |  |  |
| Total manifestations meeting WHO criteria | **7,157** | **3751**^9^ |  |  |  |
| ^1^ Numbers do not add up to total number of patients who died as there are patients with multiple manifestations  ^2^ 397 children  ^3^ Onset of respiratory manifestation was before starting antimalarial treatment in 96 patients and after in 84. Unknown onset for the remaining patients.  ^4^ Unspecified hepatic dysfunction or raised enzymes  ^5^ Cardiac, haemophagocytic syndrome, rhabdomyolysis, hydrocephalus, unspecified bleeding, cholecystitis  ^6^  Unspecified manifestation including WHO definition (n=180)  ^7^ Total number of patients died as a numerator. The total number of patients diagnosed with vivax malaria in the included studies is 1,059,970  ^8^ There are patients with more than one severe manifestation.  ^9^  Including unspecified manifestation with WHO definition (n=27)  *28 and **97 patients with bleeding.  ^†^25 aggregated with Jarzan (Saudi), ^††^3 aggregated with Manaus (Brazil) | | | | | |

**List of abbreviations:**

ADEM Acute disseminated encephalomyelitis

AKI Acute kidney injury

ARDS Acute respiratory distress syndrome

BUN Blood urea nitrogen

Cre Creatinine

CXR Chest radiograph

DIC Disseminated intravascular coagulation

GCS Glasgow Coma Score

Hb Haemoglobin

KDIGO Kidney Disease: Improving Global Outcomes

MODS Multi-organ Dysfunction Syndrome

PMNS Post-malaria neurological syndrome

RIFLE Risk, Injury, Failure; Loss, End-Stage Renal Disease

RR Respiratory rate

WHO World Health Organization
