# Supplementary material for: Clinical impact of vivax malaria: A collection review
Source: PLoS Med. 2022 Jan 18;19(1):e1003890. doi: 10.1371/journal.pmed.1003890 (PMC8765657; doi:10.1371/journal.pmed.1003890)
Supplement: S6 Table — (DOCX) [file pmed.1003890.s007.docx]

**S6a Table: Assessment of small study effects in estimation of proportion of patients with severe vivax malaria**

|  |  |  | **Estimates derived from meta-analysis of proportion** | | | **Sensitivity analysis** | |
| --- | --- | --- | --- | --- | --- | --- | --- |
| **Definition of severe malaria** | **Number of articles ^a^** | **n/N** | **Fixed effect**  **[95% confidence interval]** | ***I*^2^** | **Random effects**  **[95% confidence interval]** | **Test for funnel plot asymmetry ^d^** | **Estimated adjusted for small study effects using trim-and-fill method** |
| **WHO definition** |  |  |  |  |  |  |  |
| Overall | 84 | 4,518 / 818,808 | 0.55% [0.54%–0.57%] | 99.6% | 4.37% [2.60%–7.25%] | *P*<0.001 | 0.85% [0.45%–1.58%] ^e^ |
| **Stratified by region** |  |  |  |  |  |  |  |
| Africa | 5 | 73 / 3,306 | 2.21% [1.76%–2.77%] | 93.7% | 1.62% [0.18%–12.94%] | - | - |
| Asia | 66 | 3,572 / 330,131 | 1.08% [1.05%–1.12%] | 99.5% | 7.29% [4.47%–11.67%] | 0.002 | 3.61% [2.15%–5.99%] ^e^ |
| Oceania | 1 | 16 / 1,946 | 0.82% [0.50%–1.34%] | - | 0.82% [0.50%–1.34%] | - | - |
| South America | 13 | 857 / 483,425 | 0.18% [0.17%–0.19%] | 99.2% | 0.58% [0.12%–2.87%] | 0.084 | - |
| **Stratified by settings** |  |  |  |  |  |  |  |
| Hospitalised | 66 | 2,594 / 35,822 | 7.24% [6.98%–7.51%] | 97.1% | 7.11% [4.3%–11.55%] | 0.004 | 20.55% [12.93%–31.06%] |
| All (including outpatients) ^b^ | 14 | 1,836 / 780,960 | 0.24% [0.22%–0.25%] | 99.5% | 0.70% [0.19%–2.57%] | 0.051 | 0.35% [0.09%–1.30%] ^e^ |
| Other ^c^ | 4 | 88 / 2,026 | 4.34% [3.54%–5.32%] | 93.9% | 1.92% [0.26%–12.66%] | - | - |
| **All definition** |  |  |  |  |  |  |  |
| Overall | 82 | 6,063 / 818,541 | 0.74% [0.72%–0.76%] | 99.7% | 11.53% [8.09%–16.18%] | *P*<0.001 | 1.75% [0.97%–3.12%] ^e^ |
| **Stratified by region** |  |  |  |  |  |  |  |
| Africa | 5 | 202 / 3,306 | 6.11% [5.34%–6.98%] | 97.9% | 9.69% [3.54%–23.84%] | 0.668 | - |
| Asia | 64 | 4,226 / 329,864 | 1.28% [1.24%–1.32%] | 99.6% | 16.02% [11.46%–21.94%] | *P*<0.001 | 4.42% [2.65%–7.28%] ^e^ |
| Oceania | 1 | 100 / 1,946 | 5.14% [4.24%–6.21%] | - | 5.14% [4.24%–6.21%] | - | - |
| South America | 13 | 1,535 / 483,425 | 0.32% [0.30%–0.33%] | 99.5% | 2.24% [0.72%–6.78%] | 0.023 | 0.41% [0.10%–1.69%] |
| **Stratified by settings** |  |  |  |  |  |  |  |
| Hospitalised | 65 | 3,420 / 3,5742 | 9.57% [9.27%–9.80%] | 97.5% | 17.74% [13.19%–23.42%] | 0.346 | - |
| All (including outpatients) ^b^ | 14 | 2,560 / 780,960 | 0.33% [0.32%–0.34%] | 99.6% | 1.57% [0.61%–4.00%] | 0.010 | 0.47% [0.14%–1.49%] |
| Other ^c^ | 3 | 83 / 1,839 | 4.51% [3.65%–5.56%] | 96.8% | 5.06% [1.18%–19.22%] | - | - |

WHO World Health Organization

n= Number of patients with severe vivax malaria; N = Total number of patients with vivax malaria; ^a^ Studies that were carried out exclusively among pregnant women are excluded; studies that include few or some pregnant women were not excluded; ^b^ Studies that predominantly reported data on outpatients settings were also included; ^c^ Other includes studies that did not mention the settings and the studies in which the number of patients who were hospitalised or treated outpatients could not be reliably extracted; ^d^ Only carried when there were ≥10 studies included in the meta-analysis using Egger test using metabias function in R meta library; ^e^ The corresponding estimates derived using copas selection model using copas function in R metasens library led to warnings regarding Hessian matrix and hence estimates derived from only trim-and-fill method is presented

**S6b Table: Assessment of small study effects in estimation of mortality in studies eligible for inclusion in the meta-analysis**

|  |  |  | **Estimates derived from meta-analysis of proportion** | | | **Sensitivity analysis** | |
| --- | --- | --- | --- | --- | --- | --- | --- |
|  | **Number of**  **articles ^a^** | **n/N** | **Fixed effect**  **[95% confidence interval]** | ***I*^2^** | **Random effects**  **[95% confidence interval]** | **Test for funnel plot asymmetry ^e^** | **Estimated adjusted for small study effects using trim-and-fill method** |
| Overall | 75 | 334/814,505 | 0.04% [0.04%–0.05%] | 96.2% | 0.27% [0.15%–0.5%] | 0.373 | 0.06% [0.03%–0.12%]^f^ |
| **Stratified by region** |  |  |  |  |  |  |  |
| Africa | 2 | 0/2,263 | 0.00% [0.00%–0.17%]^d^ | - | 0.00% [0.00%–0.17%]^d^ | - | - |
| Asia | 62 | 271/329,257 | 0.08% [0.07%–0.09%] | 93.8% | 0.50% [0.29%–0.84%] | 0.701 | - |
| Oceania | - | - | - | - | - | - | - |
| South America | 12 | 63/482,985 | 0.01% [0.01%–0.02%] | 95.8% | 0.03% [0.00%–0.24%] | 0.122 | - |
| **Stratified by settings** |  |  |  |  |  |  |  |
| Hospitalised | 61 | 234/36,087 | 0.65% [0.57%–0.74%] | 80.6% | 0.56% [0.35%–0.92%] | 0.023 | 2.19% [1.5%–3.18%] |
| All (including outpatients) ^b^ | 11 | 82/778,041 | 0.01% [0.01%–0.01%] | 95.2% | 0.01% [0.00%–0.07%] | 0.353 | - |
| Other ^c^ | 3 | 18/377 | 4.77% [3.03%–7.45%] | 75.6% | 2.50% [0.59%–9.98%] | - | - |

n= Number of patients who died; N = total number of patients with vivax malaria; ^a^ Studies that were carried out exclusively among pregnant women are excluded; studies that include few or some pregnant women were not excluded; ^b^ Studies that predominantly reported data on outpatients settings were also included; ^c^ Other includes studies that did not mention the settings and the studies in which the number of patients who were hospitalised or treated outpatients could not be reliably extracted; ^d^ 95% confidence interval were obtained using Wilson’s method ignoring the site effects; ^e^ Only carried when there were ≥10 studies included in the meta-analysis using Egger test using metabias function in R meta library; ^f^ The corresponding estimates derived using copas selection model using copas function in R metasens library led to warnings regarding Hessian matrix and hence estimates derived from only trim-and-fill method is presented

**S6c Table: Assessment of small study effects in estimation of proportion of patients with cerebral malaria, renal complications and respiratory complications in studies eligible for inclusion in the meta-analysis**

|  |  |  | **Estimates derived from meta-analysis of proportion** | | | **Sensitivity analysis** | |
| --- | --- | --- | --- | --- | --- | --- | --- |
|  | **Number of**  **articles ^a^** | **n/N** | **Fixed effect**  **[95% confidence interval]** | ***I*^2^** | **Random effects**  **[95% confidence interval]** | **Test for funnel plot**  **asymmetry ^d^** | **Estimated adjusted for small study effects using trim-and-fill method** |
| **Cerebral malaria** |  |  |  |  |  |  |  |
| **WHO definition** |  |  |  |  |  |  |  |
| Overall | 90 | 636/820,671 | 0.08% [0.07%–0.08%] | 97.7% | 0.28% [0.14%–0.56%] | 0.668 | - |
| Hospitalised | 71 | 466/37,602 | 1.24% [1.13%–1.36%] | 89.8% | 0.62% [0.33%–1.18%] | *P*<0.001 | 5.18% [3.22%–8.20%]^e^ |
| All (including outpatients) ^b^ | 14 | 156/780,960 | 0.02% [0.02%–0.02%] | 98.3% | 0.02% [0.00%–0.13%] | 0.571 | - |
| Other ^c^ | 5 | 14/2,109 | 0.66% [0.39%–1.12%] | 80.9% | 0.10% [0.00%–3.68%] | - | - |
| **All definitions** |  |  |  |  |  |  |  |
| Overall | 90 | 962/820,671 | 0.12% [0.11%–0.12%] | 98.3% | 0.83% [0.51%–1.35%] | 0.153 | - |
| Hospitalised | 71 | 631/37,602 | 1.68% [1.55%–1.81%] | 91.2% | 1.43% [0.90%–2.25%] | *P*<0.001 | 5.88% [3.87%–8.82%] |
| All (including outpatients) ^b^ | 14 | 310/780,960 | 0.04% [0.04%–0.04%] | 98.5% | 0.10% [0.03%–0.32%] | 0.311 | - |
| Other ^c^ | 5 | 21/2,109 | 1.00% [0.65%–1.52%] | 84.1% | 0.87% [0.13%–5.69%] |  |  |
| **Renal** |  |  |  |  |  |  |  |
| **WHO definition** |  |  |  |  |  |  |  |
| Overall | 89 | 447/820,351 | 0.05% [0.05%–0.06%] | 89.7% | 0.18% [0.08%–0.39%] | *P*<0.001 | 3.77% [2.12%–6.60%] |
| Hospitalised | 70 | 405/37,282 | 1.09% [0.99%–1.2%] | 87.7% | 0.51% [0.26%–0.98%] | *P*<0.001 | 4.40% [2.75%–6.96%] ^e^ |
| All (including outpatients) ^b^ | 14 | 39/780,960 | 0.00% [0.00%–0.01%] | 91.2% | 0.01% [0.00%–0.10%] | 0.001 | 1.04% [0.11%–9.16%] ^e^ |
| Other ^c^ | 5 | 3/2,109 | 0.14% [0.05%–0.44%] | 0.0% | 0.14% [0.05%–0.44%] | - | - |
| **All definitions** |  |  |  |  |  |  |  |
| Overall | 88 | 810/820,248 | 0.1% [0.09%–0.11%] | 95.0% | 0.49% [0.25%–0.97%] | *P*<0.001 | 7.04% [3.81%–12.65%] |
| Hospitalised | 69 | 759/37,179 | 2.04% [1.9%–2.19%] | 94.1% | 1.29% [0.73%–2.26%] | *P*<0.001 | 8.52% [5.12%–13.83%] ^e^ |
| All (including outpatients) ^b^ | 14 | 48/780,960 | 0.01% [0.00%–0.01%] | 93.0% | 0.02% [0.00%–0.19%] | 0.013 | 0.79% [0.09%–6.24%] |
| Other ^c^ | 5 | 3/2,109 | 0.14% [0.05%–0.44%] | 0.0% | 0.14% [0.05%–0.44%] | - | - |
| **Respiratory** |  |  |  |  |  |  |  |
| **WHO definition** |  |  |  |  |  |  |  |
| Overall | 87 | 414/819,669 | 0.05% [0.05%–0.06%] | 92.6% | 0.23% [0.11%–0.47%] | 0.002 | 3.55% [2.05%–6.07%] |
| Hospitalised | 69 | 352/37,233 | 0.95% [0.85%–1.05%] | 86.6% | 0.66% [0.38%–1.13%] | *P*<0.001 | 4.03% [2.60%–6.20%] |
| All (including outpatients) ^b^ | 13 | 57/780,327 | 0.01% [0.01%–0.01%] | 85.5% | 0.00% [0.00%–0.07%] | 0.435 | - |
| Other ^c^ | 5 | 5/2,109 | 0.24% [0.1%–0.57%] | 55.0% | 0.27% [0.06%–1.16%] | - | - |
| **All definitions** |  |  |  |  |  |  |  |
| Overall | 87 | 848/819,669 | 0.10% [0.10%–0.11%] | 98.1% | 0.66% [0.41%–1.05%] | 0.040 | 0.11% [0.06%–0.2%] ^e^ |
| Hospitalised | 69 | 450/37,233 | 1.21% [1.10%–1.32%] | 90.4% | 1.00% [0.62%–1.61%] | *P*<0.001 | 5.24% [3.36%–8.09%] ^e^ |
| All (including outpatients) ^b^ | 13 | 393/780,327 | 0.05% [0.05%–0.06%] | 98.8% | 0.18% [0.06%–0.53%] | 0.296 | - |
| Other ^c^ | 5 | 5/2,109 | 0.24% [0.10%–0.57%] | 55.0% | 0.27% [0.06%–1.16%] |  |  |

WHO World Health Organization

n= Number of patients with the given complications; N = total number of patients with vivax malaria; ^a^ Studies that were carried out exclusively among pregnant women are excluded; studies that include few or some pregnant women were not excluded; ^b^ Studies that predominantly reported data on outpatients settings were also included; ^c^ Other includes studies that did not mention the settings and the studies in which the number of patients who were hospitalised or treated outpatients could not be reliably extracted; ^d^ Only carried when there were ≥10 studies included in the meta-analysis using Egger test using metabias function in R meta library; ^e^ The corresponding estimates derived using copas selection model using copas function in R metasens library led to warnings regarding Hessian matrix and hence estimates derived from only trim-and-fill method is presented
