# Supplementary material for: Clinical impact of vivax malaria: A collection review
Source: PLoS Med. 2022 Jan 18;19(1):e1003890. doi: 10.1371/journal.pmed.1003890 (PMC8765657; doi:10.1371/journal.pmed.1003890)
Supplement: S7 Table — (DOCX) [file pmed.1003890.s008.docx]

**S7 Table: Studies of severe vivax malaria in pregnant women only**

|  | Number of articles | n/N | Fixed effect  [95% confidence interval] | *I*^2^ | Random effects  [95% confidence interval] |
| --- | --- | --- | --- | --- | --- |
| **Severe vivax malaria** |  |  |  |  |  |
| WHO definition | 4 | 10/225 | 4.44% [2.41%–8.06%] | 0% | 4.14% [0.82%–18.43%] |
| All definitions | 4 | 26/225 | 11.56% [7.99%–16.43%] | 76% | 12.16% [5.95%–23.26%] |
|  |  |  |  |  |  |
| **Mortality** | 4 | 0/225 | 0% ^a^ | - | 0% ^a^ |
| **Cerebral malaria** |  |  |  | - |  |
| WHO definition | 4 | 0/225 | 0% ^a^ |  | 0% ^a^ |
| All definitions | 4 | 0/225 | 0% ^a^ |  | 0% ^a^ |
| **Renal** |  |  |  |  |  |
| WHO definition | 4 | 0/225 | 0% ^a^ |  | 0% ^a^ |
| All definitions | 4 | 0/225 | 0% ^a^ |  | 0% ^a^ |
| **Respiratory** |  |  |  |  |  |
| WHO definition | 4 | 0/225 | 0% ^a^ |  | 0% ^a^ |
| All definitions | 4 | 0/225 | 0% ^a^ |  | 0% ^a^ |

WHO World Health Organization

n= Number of patients with the given outcome; N = total number of patients with vivax malaria; ^a^ Meta-analysis not carried out as there were no events
