# Supplementary material for: Clinical impact of vivax malaria: A collection review
Source: PLoS Med. 2022 Jan 18;19(1):e1003890. doi: 10.1371/journal.pmed.1003890 (PMC8765657; doi:10.1371/journal.pmed.1003890)
Supplement: S8 Table — (DOCX) [file pmed.1003890.s009.docx]

**S8a Table: Estimates of proportion of severe vivax patients stratified by settings within each regions**

|  | Number of articles ^a^ | n/N | Fixed effect [95% confidence interval] | I^2^ | Random effects [95% confidence interval] | Test for difference between settings using random effects model ^d^ |
| --- | --- | --- | --- | --- | --- | --- |
| **Africa** |  |  |  |  |  |  |
| **All definition** |  |  |  |  |  |  |
| Hospitalised | 4 | 147/2773 | 5.3% [4.53%–6.2%] | 98.4% | 9.59% [2.68%–28.97%] | *P*=0.9073 |
| All (including outpatients) ^b^ | 1 | 55/533 | 10.32% [8.01%–13.2%] | - | 10.32% [8.01%–13.2%] |  |
| Other ^c^ | - | - | - | - | - |  |
| **WHO definition** |  |  |  |  |  |  |
| Hospitalised | 4 | 18/2773 | 0.65% [0.41%–1.03%] | 93.7% | 0.91% [0.06%–11.95%] | *P*=0.0672 |
| All (including outpatients) ^b^ | 1 | 55/533 | 10.32% [8.01%–13.2%] | - | 10.32% [8.01%–13.2%] |  |
| Other ^c^ | - | - | - | - | - |  |
| **Asia** |  |  |  |  |  |  |
| **All definition** |  |  |  |  |  |  |
| Hospitalised | 56 | 3159/3196 | 9.88% [9.56%–10.22%] | 97.6% | 19.35% [14.02%–26.1%] | *P*=0.0014 |
| All (including outpatients) ^b^ | 5 | 984/296061 | 0.33% [0.31%–0.35%] | 99.6% | 3.23% [1.06%–9.41%] |  |
| Other ^c^ | 3 | 83/1839 | 4.51% [3.65%–5.56%] | 96.8% | 5.06% [1.18%–19.22%] |  |
| **WHO definition** |  |  |  |  |  |  |
| Hospitalised | 57 | 2500/32044 | 7.8% [7.51%–8.1%] | 97.4% | 8.55% [5.05%–14.12%] | *P*=0.1221 |
| Outpatients | 5 | 984/296061 | 0.33% [0.31%–0.35%] | 99.6% | 3.23% [1.06%–9.41%] |  |
| Other | 4 | 88/2026 | 4.34% [3.54%–5.32%] | 93.9% | 1.92% [0.26%–12.66%] |  |
| **South America** |  |  |  |  |  |  |
| **All definition** |  |  |  |  |  |  |
| Hospitalised | 6 | 114/1005 | 11.34% [9.53%–13.46%] | 90.0% | 11.53% [6.26%–20.26%] | *P*<0.0001 |
| All (including outpatients) ^b^ | 7 | 1421/482420 | 0.29% [0.28%–0.31%] | 99.4% | 0.58% [0.15%–2.25%] |  |
| Other ^c^ | - | - | - | - | - |  |
| **WHO definition** |  |  |  |  |  |  |
| Hospitalised | 6 | 76/1005 | 7.56% [6.08%–9.37%] | 63.5% | 5.52% [2.43%–12.04%] | *P*=0.0009 |
| All (including outpatients) ^b^ | 7 | 781/482420 | 0.16% [0.15%–0.17%] | 98.5% | 0.12% [0.01%–1.01%] |  |
| Other ^c^ | - | - | - | - | - |  |
| **Oceania** ^e^ |  |  |  |  |  |  |
| **All definition (outpatients)** | 1 | 100/1946 | 5.14% [4.20%–6.22%] | - | 5.14% [4.20%–6.22%] | - |
| **WHO definition** | 1 | 16/1946 | 0.82% [0.47%–1.33%] | - | 0.82% [0.47%–1.33%] | - |

WHO World Health Organization

n= Number of patients with severe vivax malaria; N = Total number of patients with vivax malaria;

^a^ Studies that were carried out exclusively among pregnant women are excluded; studies that include few or some pregnant women were not excluded;

^b^ Studies that predominantly reported data on outpatients settings were also included;

^c^ Other includes studies that did not mention the settings and the studies in which the number of patients who were hospitalised or treated outpatients could not be reliably extracted

^d^ The *P*-value reported is for the comparison of difference between three settings within each region for the given definition of severe vivax malaria. Example: *P*=0.9073 is for the comparison of differences between settings in Africa using all definition of severe vivax malaria.

^e^ Data available from only one study and 95% confidence interval presented is from Clopper-Pearson method

**Investigation of interaction between settings and regions**

The following grouped logistic regression models with study as random effects were fitted to explore interaction between region and settings. When interaction term between region and setting was included in the model, the fixed-effect model matrix was rank deficient with warnings of model convergence. Therefore, some of the categories of the two factors were combined (Oceania and Asia were grouped together) and the other and outpatients settings were grouped together.

**S8b Table: Grouped logistic regression fitted**

| Model | Model formulation |
| --- | --- |
| Model 0 | Intercept only |
| Model 1 | Settings |
| Model 2 | Region |
| Model 3 | Region + Settings (Main effects only) |
| Model 4 | Region*Settings (Model with Interaction) |

For each definition of severe vivax malaria, the specified five different models were fitted. Model comparisons was based on the changes in the log-likelihood of the models using chi-squared test and is presented below separately for All definition and the WHO definition.

R script used for generating the results is available from: <https://github.com/PrabinDahal/severe-vivax-review>

**S8c Table: Comparison of model fit between nested models**

|  | All definition of  severe vivax malaria | | | WHO definition of  severe vivax malaria | | |
| --- | --- | --- | --- | --- | --- | --- |
|  | X^2^ statistics | Degrees of freedom | *P*-value | X^2^ statistics | Degrees of freedom | *P*-value |
| Model 1 vs Model 0 | 61.55 | 2 | *P*<0.001 | 57.47 | 2 | *P*<0.001 |
| Model 2 vs Model 0 | 27.30 | 1 | *P*<0.001 | 12.67 | 1 | 0.0003 |
| Model 3 vs Model 1 | 20.64 | 1 | *P*<0.001 | 9.43 | 1 | 0.00214 |
| Model 3 vs Model 2 | 54.89 | 2 | *P*<0.001 | 54.22 | 2 | *P*<0.001 |
| Model 4 vs Model 3 | 2.56 | 2 | 0.2782 | 5.03 | 2 | 0.0808 |

**S8d Table: Output of model 3**

The pairwise comparison between Outpatients settings against other region was statistically significant (P<0.001) whereas the comparison between regions was not significant (Asia vs Africa: *P*=0.054; South America vs Africa: *P*=0.543). Partial results from the Model 3 is produced below:

Random effects:

Groups Name Variance Std.Dev.

unique_ID (Intercept) 2.106 1.451

Number of obs: 83, groups: unique_ID, 82

Fixed effects:

Estimate Std. Error z value Pr(>|z|)

(Intercept) -1.8240 0.6640 -2.747 0.00601 **

region_1 Asia 0.4134 0.6805 0.607 0.54356

region_1 South America -0.8648 0.6974 -1.240 0.21495

settings_1 outpatients -1.9723 0.4064 -4.854 1.21e-06 ***

**Conclusions:** For either definitions of severe vivax malaria:

- Adjusting for settings and regions led to a significantly improved model fit compared to univariable models that included either region or settings
- In multivariable model that included settings and region (Model 3), the pairwise differences between settings remained significant whereas the differences between the regions was not significant
- Adding interaction between region and settings did not improve the model fit (S8c Table)
