# Supplementary material for: Clinical impact of vivax malaria: A collection review
Source: PLoS Med. 2022 Jan 18;19(1):e1003890. doi: 10.1371/journal.pmed.1003890 (PMC8765657; doi:10.1371/journal.pmed.1003890)
Supplement: S9 Table — (DOCX) [file pmed.1003890.s010.docx]

**Supplementary Table 9. Histology results of renal biopsies taken from patients with Acute Kidney Injury and vivax malaria monoinfection**

| First author | Year of publication | Site, country | N | Biopsy results | Primaquine treatment |
| --- | --- | --- | --- | --- | --- |
| Kute, V. B.(1) | 2012 | Karachi, Pakistan | 1 | Cortical necrosis, loss of cellularity. Tubular degeneration. Diffuse leucocytic interstitial infiltrate. Hemozoin negative. | Yes (after haemodialysis but before biopsy) |
| Lacerda, M.(2) | 2012 | Brazil | 1 | Acute tubular necrosis (ATN), tubular hemoglobin casts | Yes (triggered haemolysis and AKI) |
| Kaushik, R.(3) | 2013 | Uttarakhand, India | 2 | 1. ATN 2. ATN + mesangioproliferative glomerulonephritis | On recovery |
| Sinha, A.(4) | 2013 | New Delhi, India | 9 | 1. ATN (n=2) 2. ATN + mesangioproliferative glomerulonephritis (n=3) 3. ATN + cortical necrosis (n=3) 4. ATN + mesangioproliferative glomerulonephritis + cortical necrosis (n=1)   Interstitial nephritis of varying severity in all cases | No |
| Patel, M. P.(5) | 2013 | Ahmedabad, Gujarat, India | 1 | Mesangioproliferative glomerulonephritis with crescentic glomerulonephritis | No |
| Kanodia, K. V.(6) | 2013 | Ahmedabad, Gujarat, India | 1 | Enlarged glomeruli + lobular accentuation with uniform mesangial prominence. Segmental endocapillary proliferation | Yes- after recovery |
| Nayak, K. C.(7) | 2014 | Bikaner, Rajasthan, India | 2 | 1. ATN 2. ATN + mesangioproliferative glomerulonephritis | Not stated |
| Kumar, R.(8) | 2014 | Jaipur, Rajasthan, India | 1 | Cortical & glomerular necrosis, mesangial hypercellular, tubules show RBC/cast, coagulative necrosis with interstitial infiltration of mononuclear cells | Not stated |
| Keskar, V. S.(9) | 2014 | Mumbai,  Maharashtra, India | 1 | Thrombotic microangiopathy, hypercellular glomeruli with thickened capillary walls. | No |
| Naqvi, R.(10) | 2015 | Karachi, Pakistan | 14 | 1. ATN (n=6) 2. Cortical necrosis (n=4) 3. Tubulointerstitial nephritis (n=2) 4. Crescentic glomerulonephritis (n=2) | Not stated |
| Jhorawat,R.(11) | 2015 | Jaipur, Rajasthan, India | 1 | Chronic thrombotic microangiopathy | No |
| Bhadauria, D.(12) | 2017 | Lucknow, Uttar Pradesh, India | 4 | Thrombotic microangiopathy | Not stated |
| Nair, R. K.(13) | 2019 | New Delhi, India | 1 | Thrombotic microangiopathy with patchy cortical necrosis. ANA, Anti-ds DNA, ANCA, anti-cardiolipin) all negative. C3/C4 normal | Yes (after dialysis but before biopsy) |
| Agrawal, P.(14) | 2019 | Chandigarh, India | 1 | Subacute thrombotic microangiopathy. Hemosiderin deposits in glomerulus, tubules & interstitium | Yes (timing unclear) |
| Kaur, C.(15) | 2020 | New Delhi, India | 5 | Acute cortical necrosis, thrombotic microangiopathy | Not stated |

ATN Acute Tubular necrosis; ANA antinuclear antibodies; ANCA antineutrophil cytoplasmic antibodies
**References**

1. Kute VB, Trivedi HL, Vanikar AV, Shah PR, Gumber MR, Patel HV, et al. Plasmodium vivax malaria-associated acute kidney injury, India, 2010-2011. Emerging infectious diseases. 2012;18(5):842-5.

2. Lacerda MV, Fragoso SC, Alecrim MG, Alexandre MA, Magalhaes BM, Siqueira AM, et al. Postmortem characterization of patients with clinical diagnosis of Plasmodium vivax malaria: to what extent does this parasite kill? Clinical infectious diseases : an official publication of the Infectious Diseases Society of America. 2012;55(8):e67-74.

3. Kaushik R, Kaushik RM, Kakkar R, Sharma A, Chandra H. Plasmodium vivax malaria complicated by acute kidney injury: experience at a referral hospital in Uttarakhand, India. Trans R Soc Trop Med Hyg. 2013;107(3):188-94.

4. Sinha A, Singh G, Bhat AS, Mohapatra S, Gulati A, Hari P, et al. Thrombotic microangiopathy and acute kidney injury following vivax malaria. Clin Exp Nephrol. 2013;17(1):66-72.

5. Patel MP, Kute VB, Gumber MR, Gera DN, Shah PR, Patel HV, et al. Plasmodium vivax malaria presenting as hemolytic uremic syndrome. Indian J Nephrol. 2013;23(1):74-5.

6. Kanodia KV, Vanikar AV, Kute VB, Trivedi HL. Plasmodium vivax malaria associated with acute post infectious glomerulonephritis. Ren Fail. 2013;35(7):1024-6.

7. Nayak KC, Kumar S, Gupta BK, Kumar S, Gupta A, Prakash P, et al. Clinical and histopathological profile of acute renal failure caused by falciparum and vivax monoinfection: an observational study from Bikaner, northwest zone of Rajasthan, India. Journal of vector borne diseases. 2014;51(1):40-6.

8. Kumar R, Bansal N, Jhorawat R, Kimmatkar PD, Malhotra V. Renal cortical necrosis: A rare complication of Plasmodium vivax malaria. Indian J Nephrol. 2014;24(6):390-3.

9. Keskar VS, Jamale TE, Hase NK. Hemolytic uremic syndrome associated with Plasmodium vivax malaria successfully treated with plasma exchange. Indian J Nephrol. 2014;24(1):35-7.

10. Naqvi R. Plasmodium Vivax causing acute kidney injury: A foe less addressed. Pak J Med Sci. 2015;31(6):1472-5.

11. Jhorawat R, Beniwal P, Malhotra V. Plasmodium vivax induced hemolytic uremic syndrome: An uncommon manifestation that leads to a grave complication and treated successfully with renal transplantation. Tropical parasitology. 2015;5(2):127-9.

12. Bhadauria D, Vardhan H, Kaul A, Sharma RK, Gupta A, Prasad N, et al. P. vivax Malaria presenting as Thrombotic Microangiopathy. The Journal of the Association of Physicians of India. 2017;65(9):28-31.

13. Nair RK, Rao KA, Mukherjee D, Datt B, Sharma S, Prakash S. Acute kidney injury due to acute cortical necrosis following vivax malaria. Saudi J Kidney Dis Transpl. 2019;30(4):960-3.

14. Agrawal P, Kumar A, Parwaiz A, Rawat A, Tiewsoh K, Nada R. Complement factor H gene polymorphisms and vivax malaria associated thrombotic microangiopathy. Saudi J Kidney Dis Transpl. 2019;30(2):540-4.

15. Kaur C, Pramanik A, Kumari K, Mandage R, Dinda AK, Sankar J, et al. Renal detection of Plasmodium falciparum, Plasmodium vivax and Plasmodium knowlesi in malaria associated acute kidney injury: a retrospective case-control study. BMC Res Notes. 2020;13(1):37.
